# Supplementary material for: COSINE-100 full dataset challenges the annual modulation signal of DAMA/LIBRA
Source: Sci Adv. 2025 Sep 3;11(36):eadv6503. doi: 10.1126/sciadv.adv6503 (PMC12407068; doi:10.1126/sciadv.adv6503)
Supplement: Supplementary file 1 — Figs. S1 to S7 Table S1 [file sciadv.adv6503_sm.pdf]

Science Advances
AAAS

Supplementary Materials for

COSINE-100 full dataset challenges the annual modulation signal of DAMA/LIBRA

Nelson Carlin *et al.*

Corresponding author: Young Ju Ko, yjkophys@jejunu.ac.kr; Hyun Su Lee, hyunsulee@ibs.re.kr; Seung Mok Lee, physmlee@gmail.com

Sci. Adv. **11**, eadv6503 (2025)
DOI: 10.1126/sciadv.adv6503

This PDF file includes:

Figs. S1 to S7
Table S1

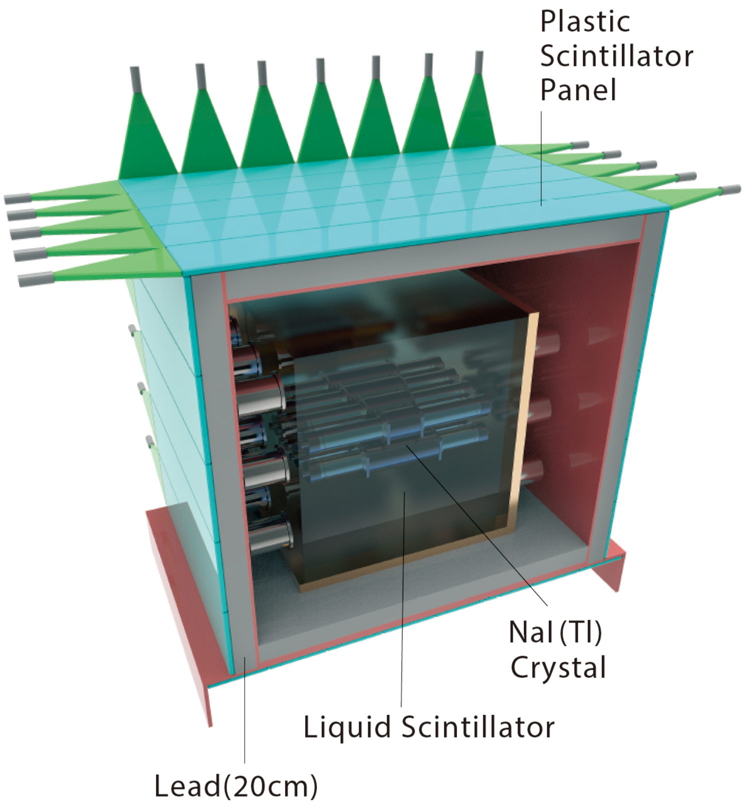

Figure S1: **Schematic of the COSINE-100 detector.** The eight encapsulated sodium iodide detectors are immersed in liquid scintillator and surrounded by 20 cm thick lead bricks and 37 plastic scintillator panels.

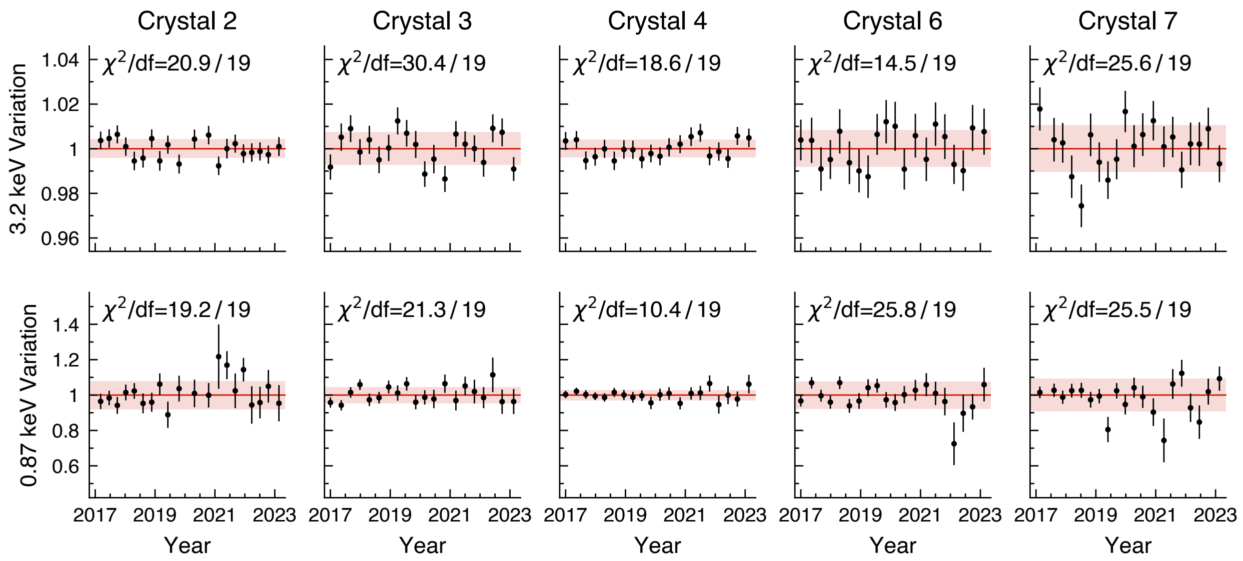

Figure S2: **Low energy calibration stability.** Time variations of 0.87 keV and 3.2 keV peak positions across each crystal are depicted. Black data points with 68.3% error bars represent measurements for different time bins, which are then divided by the overall average (red solid lines). The corresponding standard deviations are shown as filled areas. The χ^2 values calculated between the measured points and the averaged values assess the consistency and stability of these calibrations.

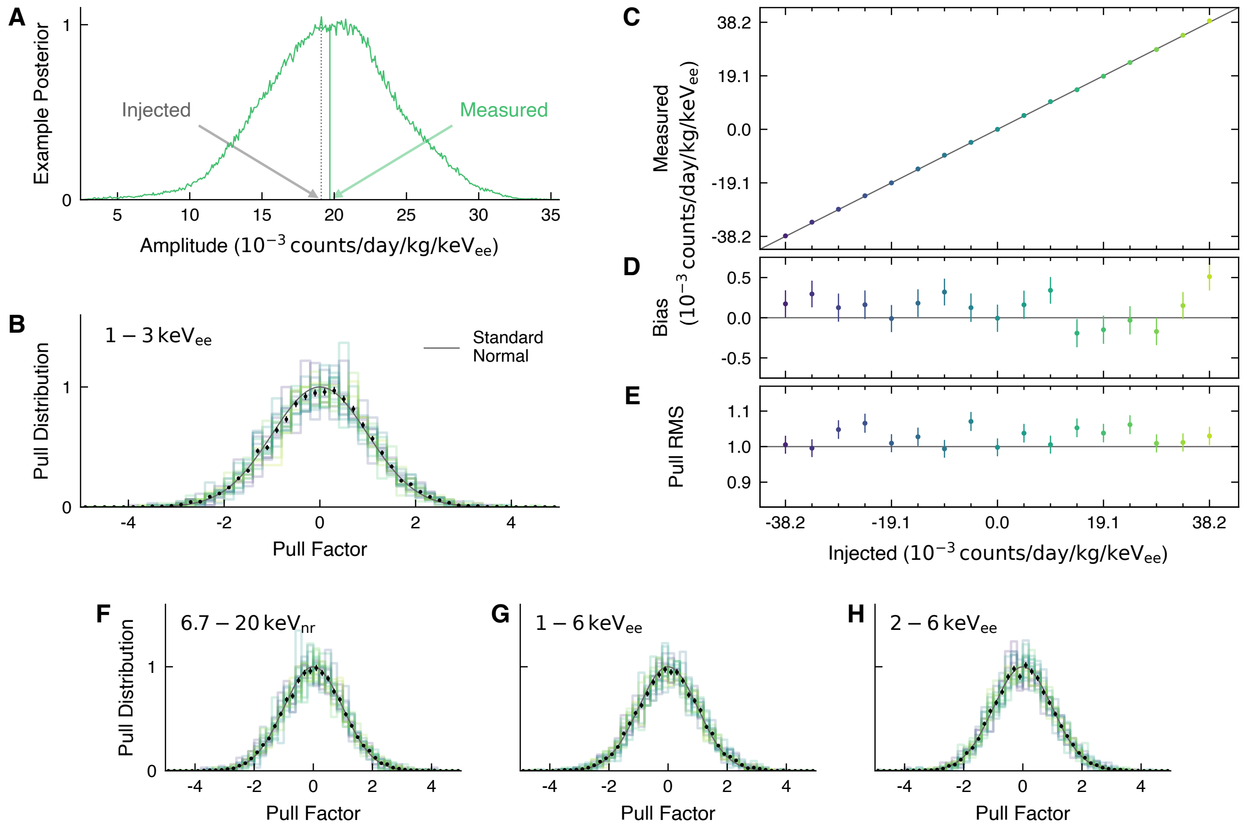

Figure S3: **Pull test in 1–3** keV$_{ee}$**.** (**A**) An example of the posterior distribution from a simulated experiment, where the DAMA/LIBRA signal was assumed. (**B**) The distribution of pull factors. Each color represents an injected modulation amplitude, and black dots represent their accumulated distribution, which is consistent with the standard normal distribution (grey solid curve). (**C**) The measured modulation amplitudes as a function of the injected amplitudes. (**D**, **E**) Bias and root-mean-square of pull factors that follow the standard normal distributions independently of the injected modulation amplitudes within the 68.3% error ranges. (**F-H**) The distributions of pull factors for different regions of interest, which are also consistent with the standard normal distribution.

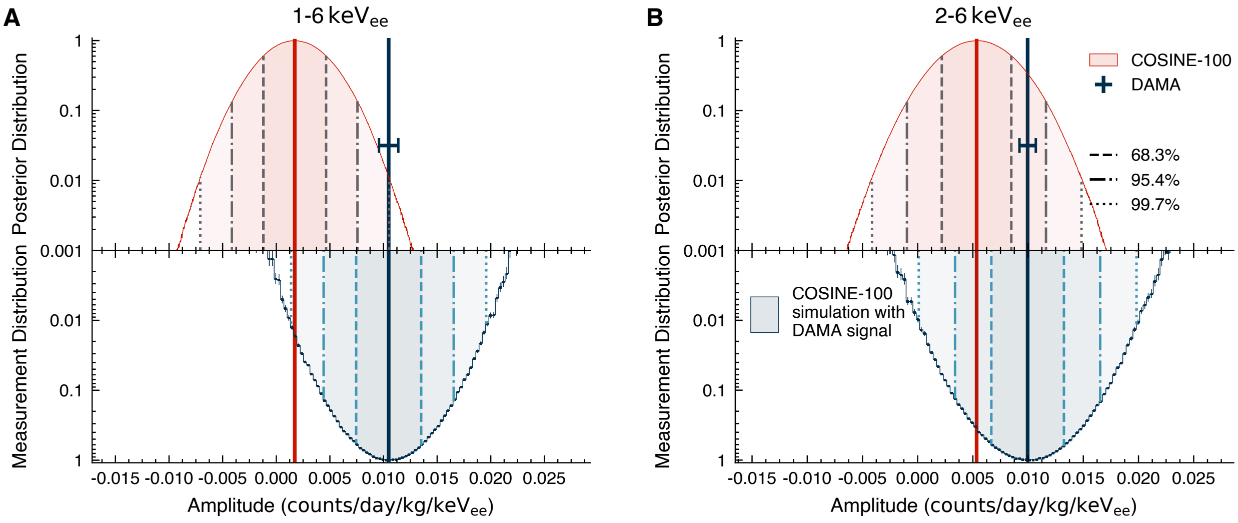

Figure S4: **Posterior distributions of modulation amplitudes from the COSINE-100 phase-fixed fits and the expected distributions for measurements assuming the DAMA/LIBRA signals.** (**A**) Modulation amplitude distribution in the 1–6 keV$_{ee}$ region. (**B**) Modulation amplitude distribution in the 2–6 keV$_{ee}$ region. The red regions represent the posterior distributions obtained from the COSINE-100 full dataset. The blue regions in the lower panels show the distributions of best-fits from simulated data, assuming the expected COSINE-100 background and the observed DAMA/LIBRA signals. The uncertainty in DAMA/LIBRA's observations has been considered during the simulation. The vertical solid lines indicate the best-fit modulation amplitudes for COSINE-100 (red) and the DAMA/LIBRA best-fit values (blue) with 68.3% errors. The other line styles indicate each probability region. The distributions are normalized to have a maximum value of unity for the comparison.

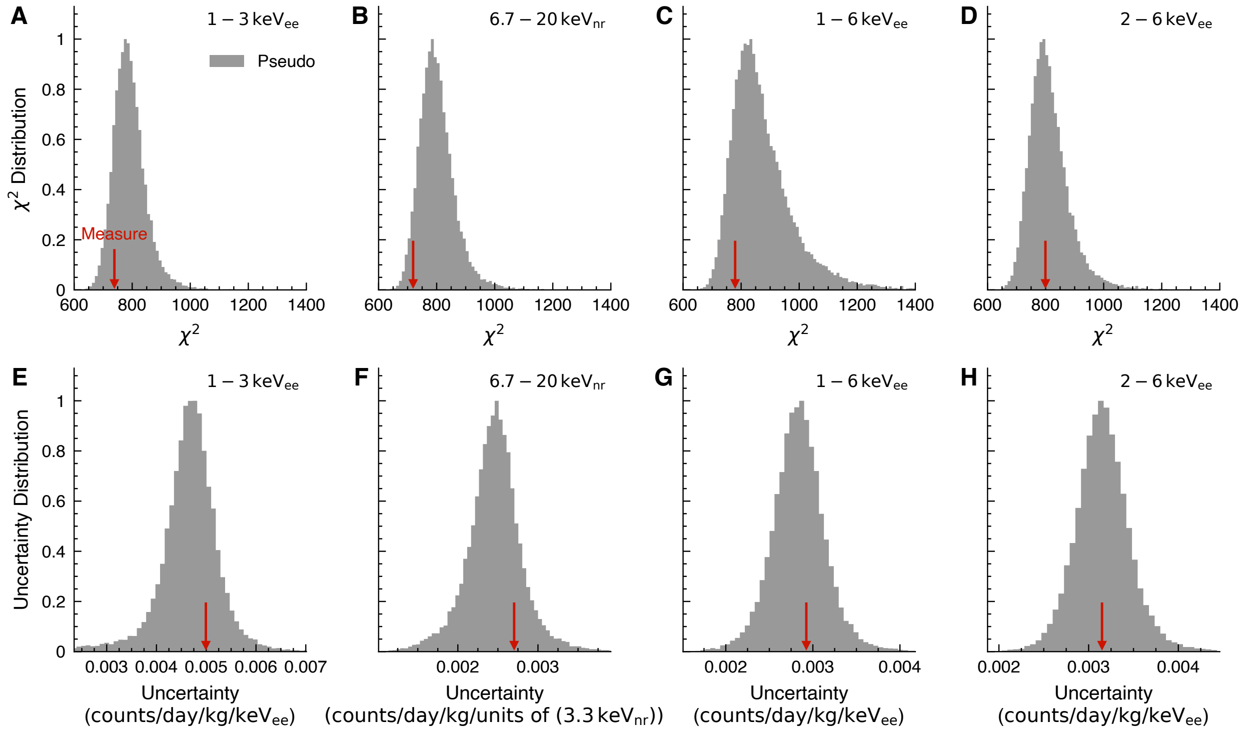

Figure S5: χ^2 **goodness-of-fits and uncertainties of the COSINE-100 data compared to the simulated experiments.** (**A–D**) χ^2s and (**E–H**) the uncertainties measured from the COSINE-100 full dataset (red arrows) are compared with distributions expected from the simulated experiments with no annual modulation signals in the considered energy ranges. In all cases, results from the COSINE-100 data are well within 2σ of the distributions from the 25,000 simulated experiments.

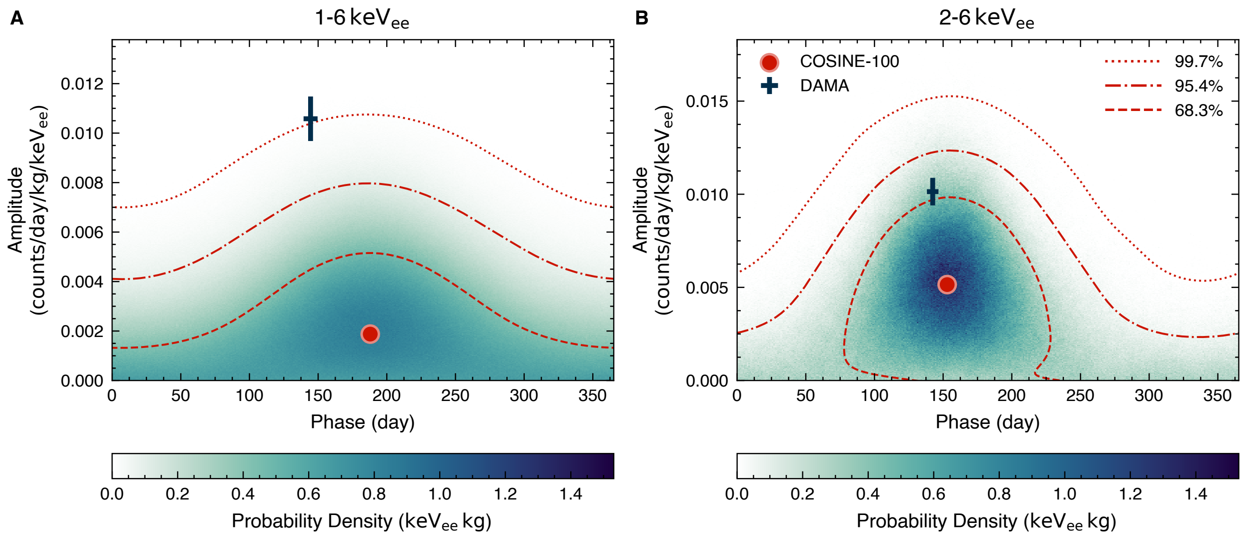

Figure S6: **Two-dimensional posterior distributions of phase-floated modulation fits for the COSINE-100 full dataset.** (**A**) Posterior distribution in the 1–6 keV$_{ee}$ region. (**B**) Posterior distribution in the 2–6 keV$_{ee}$ region. The COSINE-100 best-fit points (red dots) and the probability contours from the posterior distributions for the phase-floated fits are compared with the best-fit amplitudes and phases reported by DAMA/LIBRA (data points with 68.3% error bars).

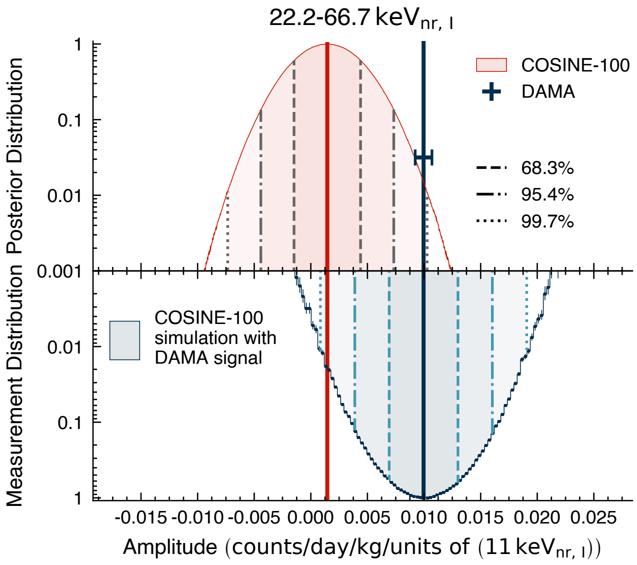

Figure S7: **Modulation amplitude distribution in the 22.2–66.7 keV$_{nr, I}$ region, considering the iodine QF difference.** The red regions represent the posterior distributions obtained from the COSINE-100 full dataset. The blue regions in the lower panels show the distributions of best-fits from simulated data, assuming the expected COSINE-100 background and the observed DAMA/LIBRA signals. The uncertainty in DAMA/LIBRA's observations has been considered during the simulation. The vertical solid lines indicate the best-fit modulation amplitudes for COSINE-100 (red) and the DAMA/LIBRA best-fit values (blue) with 68.3% errors. The other line styles indicate each probability region. The distributions are normalized to have a maximum value of unity for the comparison.

Table S1: **The expected and fitted event rates for various time-dependent background components.** The expected event rates in the 1–3 $\mathrm{keV_{ee}}$ region as of October 21, 2016, calculated from background modeling of the COSINE-100 data (*53*), are listed for different time-dependent components, together with the phase-fixed fitting results. The errors represent the 68.3% confidence intervals. Note that the flat component was not constrained during the fitting.

Component	Expected Initial Rate (counts/day/kg/$\mathrm{keV_{ee}}$)	Fitted Initial Rate (counts/day/kg/$\mathrm{keV_{ee}}$)	Half-life (years)
Total	3.67 ± 0.34	3.59 ± 0.15	
^{3}H	1.31 ± 0.32	1.33 ± 0.08	12.3
Surface ^{210}Pb	1.13 ± 0.10	1.05 ± 0.08	33.8±8.0
Internal ^{210}Pb	$(9.46 \pm 0.79) \times 10^{-1}$	$(9.13 \pm 0.72) \times 10^{-1}$	22.3
Flat	$(1.82 \pm 0.13) \times 10^{-1}$	$(1.83 \pm 0.56) \times 10^{-1}$	
^{109}Cd	$(4.48 \pm 0.65) \times 10^{-2}$	$(5.22 \pm 0.60) \times 10^{-2}$	1.26
^{127m}Te	$(2.65 \pm 0.61) \times 10^{-2}$	$(2.82 \pm 0.50) \times 10^{-2}$	0.29
^{113}Sn	$(2.04 \pm 0.39) \times 10^{-2}$	$(2.35 \pm 0.36) \times 10^{-2}$	0.31
^{22}Na	$(5.68 \pm 1.61) \times 10^{-3}$	$(6.28 \pm 1.46) \times 10^{-3}$	2.60
^{121m}Te	$(3.15 \pm 0.65) \times 10^{-3}$	$(3.24 \pm 0.61) \times 10^{-3}$	0.45
^{60}Co	$(4.38 \pm 0.28) \times 10^{-5}$	$(4.38 \pm 0.28) \times 10^{-5}$	5.27
